# Pseudo-Occlusion of the Internal Carotid Artery in Acute Ischemic Stroke: Clinical Outcome after Mechanical Thrombectomy

**DOI:** 10.1038/s41598-020-59609-9

**Published:** 2020-03-05

**Authors:** Woo Sang Jung, Jin Soo Lee, Sten Solander, Jin Wook Choi

**Affiliations:** 1 Department of Radiology, Ajou University School of Medicine, Ajou University Medical Center, Suwon, Korea; 2 Department of Neurology, Ajou University School of Medicine, Ajou University Medical Center, Suwon, Korea; 3 Department of Radiology, University of North Carolina School of Medicine, Chapel Hill, NC 27514 USA

**Keywords:** Stroke, Stroke

## Abstract

Pseudo-occlusion (PO) of the cervical internal carotid artery (cICA) can be caused by distal ICA occlusion. We explored the clinical impact of PO after mechanical thrombectomy (MT). Patients who underwent MT to treat distal ICA occlusions between July 2012 and March 2018 were reviewed. A cICA-PO was defined as when single phase computed tomography angiography (CTA) revealed a gradual decline in contrast above the level of the carotid bulb. We investigated the relationship between a cICA-PO and outcome; we also explored the association between successful recanalization and outcome. Among 71 patients, 40 (56.3%) exhibited cICA-PO and more likely to experience poor outcomes (80.0% vs. 25.8%, *P* < 0.001), hemorrhagic transformation (32.5% vs. 9.6%, *P* = 0.01), and a lower rate of successful recanalization (65.0% vs. 90.3%, *P* = 0.014) than the non-PO group. In binary logistic regression, a cICA-PO was independently associated with a poor outcome (odds ratio, 4.278; 95% CI, 1.080–33.006; *P* = 0.045). In the cICA-PO group, all patients who failed recanalization (n = 15) experienced poor outcomes, as did 69.2% of patients in whom recanalization was successful (*P* = 0.018). cICA-POs are common and have worse outcomes than non-PO patients. Patients with cICA-POs are more likely to exhibit poor outcomes after MT, particularly when recanalization fails.

## Introduction

Recent randomized controlled trials have shown that intra-arterial mechanical thrombectomy (MT) effectively and safely reduces morbidity and mortality in patients who present with acute ischemic strokes caused by large-vessel occlusions^1–5^. In the cited studies, computed tomography angiography (CTA) was most commonly used to detect and localize large artery occlusions.

A pseudo-occlusion (PO) of the cervical internal carotid artery (cICA) mimics a true occlusion in CTA but the artery is patent when digital subtraction angiography (DSA) is performed during endovascular treatment^6^. The underlying cause of a cICA-PO is sluggish or absent contrast flow caused by distal occlusion of the intracranial vasculature^7^. Several previous studies have reported that cICA-POs develop in 6–15% of patients with anterior circulation strokes and in almost 50% of those with distal ICA occlusions^7–9^. Recently, several studies have used various CTA protocols featuring delayed-phase imaging, or four-dimensional CTA after CT perfusion, to enhance diagnostic accuracy^6,8,10–13^. Here, we explored the relationships between cICA-POs in patients with acute distal ICA occlusions and the clinical outcomes after MT.

## Methods

### Study population and clinical assessment

We retrospectively reviewed consecutive patients with acute ischemic strokes who underwent reperfusion therapy between July 2012 and March 2018. Our data collection protocol was approved by the Institutional Review Board of Ajou University hospital (approval no. AJIRB-MED-MDB-18-301) and implemented in accordance with the ethical standards of the 1964 Declaration of Helsinki and its later amendments. The need for written informed consent was waived by the Institutional Review Board, given the retrospective nature of the study. We enrolled patients who underwent non-contrast CT (NCCT), CTA, DSA, catheter exploration, and MT; exhibited complete distal ICA occlusion (from the paraophthalmic to the terminal ICA segment) on DSA; and had baseline modified Rankin scale (mRS) scores ≤2. Patients for whom NCCT or CTA data were of poor quality, and those with true cICA tandem occlusions or atherosclerotic steno-occlusive changes of cICA evident in DSA, were excluded. We collected demographic, clinical, laboratory, and imaging data including age; sex; comorbidities such as hypertension, diabetes mellitus, coronary heart disease, hyperlipidemia, and/or atrial fibrillation; smoking history; time from stroke onset to puncture; time from puncture to final recanalization or angiography; National Institutes of Health Stroke Scale (NIHSS) score; hemorrhagic transformation status; 3-month mRS score; and stroke cause as determined by the Trial of ORG 10172 in Terms of the Acute Stroke Treatment (TOAST) classification.

The primary outcome parameter was the modified Rankin scale (mRS) score at 3 months after mechanical thrombectomy; a poor outcome was defined as a 3-month score of 4–6. The secondary outcome parameter was successful recanalization using the modified Thrombolysis in Cerebral Infarction (mTICI) scale (“successful” was defined as an mTICI score of 2b or 3) Hemorrhagic transformation (type 1 or 2 parenchymal hemorrhage) was identified via susceptibility-weighted magnetic resonance imaging or CT during follow-up^14^.

### Image acquisition and analyses

Our institutional stroke imaging protocol features NCCT of the head (5 mm slice thickness; 120 kV) followed by contrast-enhanced head CT (5 mm slice thickness; 120 kV). Single-phase CT angiography (100 kV, 180 mA; 10 mm slice thickness, 2 mm axial reconstruction increment) was performed using an automatic bolus-tracking technique from the aortic arch to the vertex; contrast was intravenously injected by a power injector at 5 mL/s followed by a saline flush. All CT procedures featured automated tube current modulation; we obtained multiplanar, maximum-intensity projection reformations. Endovascular procedures were performed using a biplane angiographic unit (Integris Allura; Philips Healthcare, Best, the Netherlands). An 8-F balloon-tipped guidance catheter was positioned in the common carotid artery or the proximal cICA prior to initial angiographic evaluation. Then the ICA was explored using a 0.014-inch guidewire within a 0.021-inch microcatheter. cICA patency (and hence PO status) following ICA recanalization was confirmed by complete angiography.

**Figure 1:**
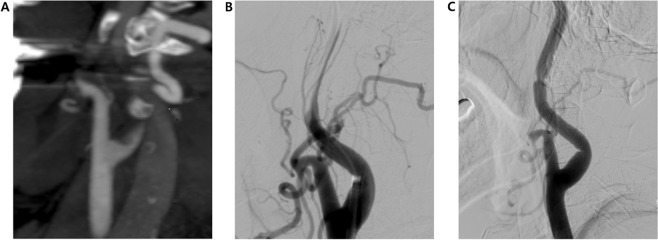
Example of a cervical ICA pseudo-occlusion on CTA with a non-corresponding cause of apparent occlusion found on DSA. (**A**) Pseudo-occlusion of the left cervical ICA in a 69-year-old woman. CTA shows a sharp, flame-shaped leading contrast edge at the level of the cervical ICA, with T-occlusion present. (**B**) DSA of this patient shows contrast moving slowly upward and finally a patent ICA (**C**) after mechanical thrombectomy of the distal ICA occlusion.

Two experienced interventional neuroradiologists independently reviewed the CTA images to detect cICA-POs, which were considered present when a gradual contrast decay (a flame-shaped leading contrast edge) was evident in the ICA above the level of the carotid bulb, in the absence of plaque around the bulb, and the vessel was normal according to angiographic exploration but a distal ICA occlusion was apparent. Subsequently, DSA images were re-examined to determine whether the cICA was in fact patent (Fig. 1). Axial angiography was used to derive collateral CTA scores of 0 to 5, based on single-phase CTA images of the ischemic and asymptomatic hemispheres: Grade 5, normal or increased prominence/extent of pial vessels; Grade 4, slightly reduced prominence/extent; Grade 3, moderately reduced prominence/extent; Grade 2, decreased prominence/extent, and regions with no vessels; Grade 1, only a few vessels; Grade 0, no vessels^15^.

### Statistical analyses

Clinical characteristics and imaging features are reported as means ± standard deviations or medians for continuous variables and as proportions for categorical variables. The independent-samples two-tailed *t*-test or the Mann–Whitney *U*-test was used (as appropriate) to compare continuous variables when the data were normally distributed; Fisher’s exact test was used to compare dichotomous variables. Parameters with *P*-values < 0.10 in univariate analyses were included in binary logistic regression. A *P*-value < 0.05 was considered to reflect statistical significance. All analyses were performed using SPSS ver. 22.0 (IBM Corp., Armonk, NY, USA).

## Results

Of 85 patients with distal ICA occlusions, we excluded 14 with true cICA tandem occlusions (n = 8), atherosclerotic steno-occlusive changes of cICA evident in DSA (n = 5), and poor-quality CTA data (n = 1). Thus, we ultimately included 71 patients with a mean age 70 ± 14 years; 41 (57.7%) were male. The median NIHSS score on admission was 19 (interquartile range 16–21). Of all patients, 56.3% (n = 40) exhibited cICA-POs combined with distal ICA occlusions. The evaluations of the two readers were in complete agreement.

**Figure 2:**
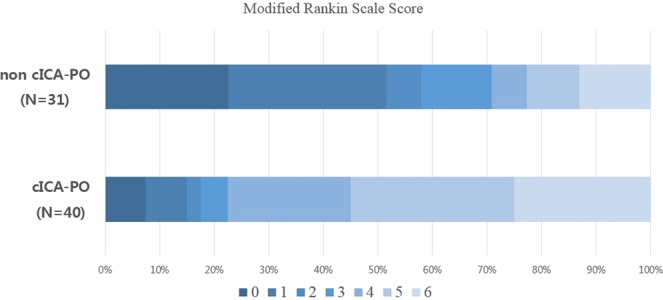
The distribution of the modified Rankin scale (mRS) scores. The scores range from 0 to 6, with 0 indicating no symptoms, 1 no clinically significant disability, 2 slight disability (patient is able to look after own affairs without assistance, but is unable to perform previous activities), 3 moderate disability (patient requires some help but is able to walk unassisted), 4 moderately severe disability (patient is unable to attend to bodily needs without assistance and unable to walk unassisted), 5 severe disability (patient requires constant nursing care and attention), and 6 death. The mRS score at 3 months according to the presence of cervical ICA pseudo-occlusion (cICA-PO). There were more patients with poor outcomes (mRS 4–6) in the cICA-PO group.

Patients with cICA-POs (n = 40) exhibited a higher rate of atrial fibrillation (82.5% vs. 58.0%, *P* = 0.024), a higher median NIHSS score on admission (19 vs. 16, *P* = 0.043), and a lower rate of successful first-pass recanalization (22.5% vs. 48.3%, *P* = 0.023), compared to non cICA-PO patients (n = 31). After MT, patients with cICA-POs exhibited a lower rate of successful recanalization (65.0% vs. 90.3%, *P* = 0.014), a higher rate of hemorrhagic transformation (32.5% vs. 9.6%, *P* = 0.01), and worse outcomes (80.0% vs. 25.8%, *P* < 0.001) than non cICA-PO patients (Fig. 2). There were no significant between-group differences in any other variable including the median ASPECTS score, median CTA collateral score, stroke onset to door time, or door to recanalization time (Table 1).

**Table 1:** Comparison of the characteristics of the cICA-PO and non-cICA-PO groups.

	Non-cICA-PO (n = 31)	cICA-PO (n = 40)	*P-*value
Baseline characteristics			
Male (%)	18 (58.0)	23 (57.5)	0.962
Age, years	66 ± 14	73 ± 13	0.037
Smoker (%)	8 (25.8)	6 (15.0)	0.26
Hypertension (%)	18 (58.0)	21 (52.5)	0.785
Diabetes mellitus (%)	9 (29.0)	8 (20.0)	0.38
Hyperlipidemia (%)	2 (6.4)	5 (12.5)	0.4
Atrial fibrillation (%)	18 (58.0)	33 (82.5)	0.024
Coronary heart disease (%)	6 (19.3)	5 (12.5)	0.432
Stroke cause			0.971
Cardioembolism (%)	26 (83.8)	34 (85.0)	
Large arterial atherosclerosis (%)	5 (16.1)	4 (10.0)	
Others (%)	0	2 (5.0)	
Intravenous rtPA, n (%)	19 (61.2)	19	0.251
Median NIHSS on admission (IQR)	16 (14–20)	19 (16–21)	0.043
Median ASPECTS (IQR)	7 (6–9)	6 (4–8)	0.137
Median CTA collateral score (IQR)	3 (3–4)	2 (1–4)	0.093
Time from onset to puncture, min	268 ± 136	411 ± 253	0.664
Time from puncture to final angiography, min	68 ± 37	85 ± 43	0.079
Outcome characteristics			
Successful recanalization (%) First-pass recanalization (%)	28 (90.3) 15 (48.3)	26 (65.0) 9 (22.5)	0.014 0.023
3-month mRS (IQR)	2 (0–3)	4 (4–6)	0.001
Poor outcome (%)	8 (25.8)	32 (80.0)	<0.001
Hemorrhagic transformation (%)	3 (9.6)	13 (32.5)	0.01

cICA-PO indicates cervical internal carotid artery pseudo-occlusion; IQR, interquartile range; others, stroke of other determined causes and undetermined causes; rtPA, recombinant tissue plasminogen activator; NIHSS, National Institutes of Health Stroke Scale; ASPECTS, Alberta Stroke Program Early CT Score; mRS, modified Rankin scale.

Binary logistic regression analyses revealed that the baseline CT ASPECTS score (odds ratio [OR], 1.605; 95% confidence interval [CI], 1.091–2.360; *P* = 0.016) and cICA-PO positivity (OR, 4.278; 95% CI, 1.080–33.006; *P* = 0.045) were independently associated with poor outcomes at 3 months (Table 2).

**Table 2:** Binary logistic regression analysis of poor outcome.

	OR	95% CI	*P*-value
HT	0.681	0.104–4.469	0.689
ASPECTS score	1.605	1.091–2.360	0.016
NIHSS score on admission	0.781	0.597–1.021	0.07
First-pass recanalization	0.66	0.124–3.505	0.625
Successful recanalization	0	0	0.998
cICA-PO	4.278	1.080–33.006	0.045
Time to puncture	0.102	0.991–1.002	0.102

CI, confidence interval; HT, hemorrhagic transformation; ASPECTS, Alberta Stroke Program Early CT Score; NIHSS, National Institutes of Health Stroke Scale; cICA-PO, cervical internal carotid artery pseudo-occlusion; OR, odds ratio.

Of the 40 patients with cICA-POs, 26 were successfully recanalized after MT, whereas 14 were not. All of the latter patients experienced poor outcomes, whereas 69.2% (n = 18) of successfully recanalized patients experienced poor outcomes (*P* = 0.018) (Table 3). Of the 71 patients, 56 exhibited proximal involvement of the M1 segment attributable to carotid-distal ICA T- or L-type occlusions; 55.4% (56/34) experienced poor outcomes. Fifteen patients had pure, distal ICA I-type occlusions lacking M1 segment involvement and nine of these 15 patients (60%) experienced poor outcomes; the prognoses did not significantly differ between the two groups (*P* = 0.747). In the PO subgroup, 29 patients evidenced M1 involvement; 22 of these 29 patients (72.5%) experienced poor outcomes. Of 11 patients lacking M1 involvement, nine (81.8%) had poor outcomes. The “poor outcome” rate did not significantly differ between the two groups (*P* = 0.687).

**Table 3:** Outcomes by recanalization status in patients with or without cICA-POs.

	No successful recanalization		Successful recanalization		*P*-value
	Total	Poor outcome	Total	Poor outcome	
cICA-PO (n = 40)	14	14 (100%)	26	18 (69.2%)	0.018
non cICA-PO (n = 31)	3	2 (66.7%)	28	6 (21.4%)	0.094

cICA-PO, cervical internal carotid artery pseudo-occlusion.

## Discussion

More than half (approximately 56%) of patients with distal ICA occlusions exhibited cICA-POs in arterial-phase CTA scans. The basic cause of PO remains unclear; the suggested mechanisms include hemodynamic insufficiency and luminal collapse triggered by acute embolism of the distal intracranial artery^16^. Patients with cICA-POs often have thrombi extending below the level of the posterior communicating artery, thus occluding that artery. Such patients tend to have larger clots (filling the terminal ICA segment) than those without cICA-POs (who usually exhibit contrast-filling of the posterior communicating artery, triggering adequate, antegrade, Willisian circle collateral flow)^7,8,12,17,18^. In other words, circle collateral flow affects cICA-PO status as determined via CTA. In addition, sluggish flow may be associated with poor collateral circulation and enlargement of the original clot, compromising successful recanalization and outcomes. Those with larger clots experience lower rates of recanalization and poorer outcomes^19^.

Endovascular treatment has become the gold standard for those with large-vessel occlusion strokes; rapid treatment and successful revascularization are important predictors of good outcomes after acute ischemic stroke^16^. Accordingly, any condition that delays or impedes recanalization negatively impacts care; it is important to image such conditions effectively. We found that those with cICA-POs tended to fail first-pass recanalization, require longer procedural times, and exhibit a lower final recanalization rate after MT than other patients. Reperfusion time was delayed, causing more brain damage. The hemorrhagic transformation rate was significantly higher in cICA-PO patients than in non cICA-PO patients (32.5% vs. 9.6%); thus, the poor outcome rate was higher in the former group. A cICA-PO in a patient with a distal ICA occlusion is a useful, surrogate imaging marker of outcome.

Patients with cICA-POs experienced lower rates of successful first-pass (22.5% vs. 48.3%) and final (65.0% vs. 90.3%) recanalization than those lacking cICA-POs. In the former group, when recanalization failed, the 3-month mRS was poor for all patients. When recanalization was successful, 69.2% of patients experienced poor outcomes; approximately 31% of patients did not. cICA-PO patients may require more aggressive approaches toward rapid recanalization.

An earlier work^13^ reported the outcomes of patients who received reperfusion therapy without mechanical thrombectomy (intravenous thrombolysis alone in 86 of 143 patients, 60.1%); all of our patients received thrombectomy. Grossberg *et al*.^9^ reported that, of 21 patients who exhibited PO on CTA, seven (33%) did not show PO on intra-arterial angiography; we evaluated patients who exhibited pseudo-occlusions on both CTA and intra-arterial catheter angiography. We found that, in the MT group, the PO sign was clinically significant. Patients with cICA-POs tended to fail first-pass recanalization, required longer procedural times, and exhibited lower final recanalization rates after MT, compared to other patients.

In patients with acute ischemic strokes associated with ipsilateral cICA non-attenuation in single-phase CTA, even specialized radiologists may not reliably distinguish true cervical occlusions from POs. In many studies, the interobserver agreement was low; CTA and DSA interpretations often differed. Our study included only cICA-POs in patients with normal DSA of the cICA. Therefore, the cICA-PO diagnostic accuracy was not a problem.

This study had some limitations. First, the single-center retrospective design of the work may feature selection bias. Second, all CT, CTA, and DSA data were interpreted simultaneously in a non-blinded manner, conceivably introducing bias. Finally, the sample size of the cICA-PO group was relatively small; we further subdivided this group into those who experienced successful and those who had non-successful recanalization. A future study should enroll more patients.

## Conclusions

cICA-PO is common in patients with acute ischemic strokes caused by distal ICA occlusion and is associated with worse outcomes compared to non-PO patients. Rapid recanalization is essential and more active approaches for obtaining complete recanalization may be required by cICA-PO patients.
